# Supplementary material for: A set of multi-entry identification keys to African frugivorous flies (Diptera, Tephritidae)
Source: Zookeys. 2014 Jul 24;(428):97–108. doi: 10.3897/zookeys.428.7366 (PMC4143993; doi:10.3897/zookeys.428.7366)
Supplement: Supplementary material 4 — Key to Capparimyia [file zookeys-428-097-s004.zip › SF4_ZooKeys_key to Capparimyia/lucid_player/help/help/whgdata/whlstf1.htm]

Search Words List


**<<**

**circumstance**   
**circumstances**   
**clean**   
click **1** **2** **3** **4** **5** **6** **7** **8** **9** **10** **11**   
clicking **1** **2** **3** **4** **5** **6** **7** **8** **9** **10**   
**closed**   
code **1** **2**   
**collapse**   
**collapsed**   
**collapsing**   
colour **1** **2**   
com **1** **2**   
**command**   
common **1** **2**   
**compare**   
**comparing**   
complete **1** **2**   
**completely**   
comprises **1** **2**   
computer **1** **2** **3** **4**   
**computers**   
**concerning**   
**configuring**   
consider **1** **2** **3**   
**considered**   
**console**   
**contain**   
**contained**   
**containing**   
contains **1** **2**   
contrast **1** **2**   
**contribute**   
control **1** **2** **3**   
**convert**   
copied **1** **2**   
copy **1** **2**   
**corals**   
correct **1** **2** **3**   
**correctly**   
**could**   
**counts**   
**course**   
**covered**   
**cream**   
create **1** **2**   
created **1** **2** **3**   
**creates**   
**crop**   
**cross**   
**crystal**   
**crystalline**   
**ctrl**   
**cubic**   
current **1** **2** **3**   
**currently**   
  
  
**danger**   
**data**   
decide **1** **2** **3**   
decimal **1** **2**   
**decrease**   
default **1** **2** **3** **4**   
**define**   
defined **1** **2**   
**depending**   
**deploy**   
deployed **1** **2** **3**   
describe **1** **2** **3** **4**   
**described**   
description **1** **2**   
**descriptions**   
designed **1** **2**   
desired **1** **2**   
**desktop**   
**details**   
determine **1** **2** **3**   
**determining**   
**developed**   
**developing**   
diagnostic **1** **2** **3**   
dialog **1** **2** **3** **4** **5** **6** **7** **8** **9**   
**dichotomous**   
differ **1** **2** **3**   
differences **1** **2** **3**   
different **1** **2**   
**difficult**   
**direct**   
**directed**   
**direction**   
discarded **1** **2** **3** **4** **5** **6** **7**   
**discarding**   
**discriminate**   
diseases **1** **2** **3** **4**   
**disorders**   
display **1** **2** **3** **4** **5** **6** **7**   
displayed **1** **2** **3** **4** **5** **6**   
displays **1** **2**   
**distinctive**   
**distributed**   
**documents**   
does **1** **2** **3** **4**   
**doesn**   
**done**   
down **1** **2** **3** **4** **5**   
download **1** **2**   
**downloaded**   
**drag**   
**dragging**   
**dropdown**   
  
  
e **1** **2**   
**e1**   
**e2**   
**e3**   
easier **1** **2** **3**   
easily **1** **2** **3** **4**   
**eastern**   
edit **1** **2**   
edition **1** **2** **3**   
**editions**   
**effective**   
**efficiently**   
either **1** **2** **3** **4**   
**elements**   
embedded **1** **2**   
**emptied**   
empty **1** **2**   
**en**   
**enable**   
enabled **1** **2**   
**encode**   
end **1** **2**   
ensure **1** **2**   
**enter**   
**entered**   
**entering**   
entities **1** **2** **3** **4** **5** **6** **7** **8** **9** **10** **11** **12** **13** **14** **15** **16** **17** **18** **19** **20** **21**   
entity **1** **2** **3** **4** **5** **6** **7** **8** **9** **10** **11**   
**entries**   
environment **1** **2**

**>>**
